# Strategies to Enhance Satisfaction and Success in an Academic Career in Pharmacoepidemiology

**DOI:** 10.1002/pds.70374

**Published:** 2026-04-15

**Authors:** Vincent Lo Re, Greta Bushnell, Luciane Cruz Lopes, Anton Pottegård

**Affiliations:** ^1^ Center for Pharmacoepidemiology and Treatment Science Rutgers University New Brunswick New Jersey USA; ^2^ Division of Infectious Diseases, Department of Medicine, Robert Wood Johnson Medical School Rutgers University New Brunswick New Jersey USA; ^3^ Department of Biostatistics and Epidemiology, School of Public Health Rutgers University New Brunswick New Jersey USA; ^4^ Pharmaceutical Sciences Graduate Course University of Sorocaba São Paulo Brazil; ^5^ Clinical Pharmacology, Pharmacy and Environmental Medicine, Department of Public Health University of Southern Denmark Odense Denmark

**Keywords:** career, mentorship, pharmacoepidemiology, reflection, vision, work‐life balance

## Abstract

**Background:**

Building an academic career in pharmacoepidemiology requires intentional planning but can be challenging, particularly for new learners, trainees, and early faculty members who may lack awareness of the skills and benchmarks expected at each career stage. This paper provides reflections on designing a purposeful and sustainable academic career.

**Perspectives:**

The first part of the article focuses on four foundational elements: (1) setting aside time for regular reflection to clarify values and priorities; (2) developing a clear vision and defining missions for each professional and personal role; (3) prioritizing activities systematically to maintain alignment with one's vision, missions, and goals; (4) seeking and maintaining mentorship throughout one's career to obtain feedback and guide decision‐making. We describe concrete strategies, tools, and authors' personal approaches for implementing these elements. The second part of the article outlines key skills to acquire and benchmarks to achieve during key career stages, including competencies such as time management, team‐building, project management, research dissemination, research grant writing, mentorship, and leadership. In the final part, we note that structural, institutional, and life‐course factors (e.g., gender, race/ethnicity, resource access, and major life events) may shape the feasibility and impact of these strategies, underscoring the importance of supportive environments, inclusive mentorship, and flexibility for sustainable careers.

**Implications:**

Our goal is not to prescribe a single pathway but to offer a reference that promotes individual reflection and catalyzes open discussion among peers, mentors, and colleagues to enhance satisfaction and success in an academic career in pharmacoepidemiology.

## Key Points

- Intentional career design is essential to build a purposeful, satisfying, and sustainable academic path in pharmacoepidemiology.
- Regular reflection to clarify core values, vision, and role‐specific missions, as well as thoughtful prioritization of activities and sustained mentorship, are critical to success in an academic career in pharmacoepidemiology.
- Structural and contextual factors shape career development, emphasizing the importance of equity, inclusive mentorship, and supportive academic environments.
- Different career stages—new learner/trainee, late trainee, early faculty, late faculty—require mastering distinct skills and achieving specific benchmarks.
- This paper is intended to foster individual reflection and stimulate dialogue between mentors, peers, and colleagues on ways to develop one's academic career in pharmacoepidemiology.

*The #1 Regret of the Dying*: I wished I had the courage to live a life true to myself …

Bronnie Ware [1]

## 1 Introduction

Building an academic career in pharmacoepidemiology can be challenging, especially at the early stages. As leaders of academic pharmacoepidemiology training programs and mentors of individuals across different stages of the career arc, we have frequently observed trainees and early faculty members who would have benefited from developing key skills at certain stages of their academic career and from having an awareness of important benchmarks for success at each stage. These individuals struggled with often unrecognized barriers that limited what they could achieve and made it difficult to balance their personal and professional lives. These challenges are not evenly distributed, with a growing body of evidence showing that structural and cultural factors such as gender, race/ethnicity, and access to resources shape opportunities and career progression in academia [2, 3, 4].

In our roles, we encourage being intentional in designing a career that is purposeful and meaningful to oneself. This requires time to reflect on core values, personal and professional priorities, and longer‐term vision and missions. It also warrants regular review of daily activities and consideration of whether these activities are aligned with individual values, missions, and priorities. We recognize that many pharmacoepidemiology training programs may not discuss how to design an academic career or convey the skills that should be acquired and benchmarks that need to be met at each stage. We believe that articulating these concepts could help to enhance career satisfaction, increase productivity, and reduce attrition from the field.

In this Commentary, we provide suggestions and individual perspectives for designing an academic career in pharmacoepidemiology and ensuring that important skills and metrics of success are considered at various time points along the path. A number of the suggestions that we offer are not necessarily specific to the field of pharmacoepidemiology, but we believe that applying these foundational elements will enhance the likelihood for career and life fulfillment and success. Some suggestions may seem immediately sensible, while others may become apparent only during certain experiences, and some may not resonate at all. We recognize that academic careers in pharmacoepidemiology are highly individualized and that approaches to career development may differ across cultures, regions, and institutions. Our goal is not to be prescriptive but rather to provide a reference to enable academic pharmacoepidemiologists to navigate each stage of their career successfully and to catalyze direct and open conversations between peers, mentors and mentees, and junior and senior colleagues within the field. We encourage readers to be selective in incorporating the suggestions provided, to experiment with implementing these suggestions, and assess whether they provide value.

We first discuss the importance of reflection to clarify core values, priorities, and the actions needed to achieve individual ambitions. We then turn to developing a personal and professional vision, defining role‐specific missions, and setting short‐ and long‐term goals. This is followed by strategies and specific tools for prioritizing activities to stay aligned with these goals. We emphasize the importance of establishing mentorship throughout an academic career to guide decision‐making and support progress. Finally, we outline key skills to develop and benchmarks to achieve at each academic stage. We include references to relevant articles and books to allow readers the opportunity to review these concepts more deeply within the primary sources. The academic environment changes over time, with the strategies we propose also being intended to support adapting to these shifts. While our focus is on academic careers, we hope these ideas will inspire similar perspective articles from pharmacoepidemiologists working in regulatory, industry, and service settings.

## 2 Setting Aside Time for Reflection

Since we have limited time and energy, it is important to be proactive and intentional in designing your career and focusing on activities and people that matter most to you [5]. However, keeping such focus in a busy academic environment can be challenging. Setting aside time for reflection can help develop and maintain your focus. Reflection is the act of purposefully examining one's thoughts, actions, and experiences to gain self‐awareness and make better choices for the future. Reflection can enhance understanding of your daily activities and effort. It allows you to consider your personal and professional priorities (i.e., the things that are most important to you right now), the priorities of your stakeholders, and the incentives and rewards that drive your decision‐making [6]. The awareness obtained through reflection can improve your decisions. The process of reflection should be repeated on a regular basis, including any time there is a major shift in your life.

### 2.1 Authors' Perspectives

Vin schedules 15 min daily for reflection, recording his thoughts in a paper journal during the quiet of the early morning. Recording his reflections allows him to collect his ideas and plans in a single place [7, 8]. He occasionally uses self‐reflective questions to prompt his thinking (see Box 1 for examples). Considering the answers to these questions has helped him to maintain focus on academic activities aligned with his missions, avoid activities that are not essential to his vision, and identify ways to improve his performance.

BOX 1 **Examples of self‐reflective questions to deepen your thinking about your personal and professional priorities and activities.**

1. How would you rate the quality of your life on a 1–10 scale with 10 as the best? Why is that your score? What would make it a 10?
2. What does a world‐class personal and professional life look like for you?
3. What needs to get done in the next 90 days, 180 days, and 360 days to feel like a success?
4. What are the 10 things that the person who is the best in the world at what you do would be doing on a regular basis?
5. What do you want people to think of when they see you coming?
6. At your funeral, what do you want loved ones to say about you? How will you wish you would have lived?
7. Imagine you are 90 years old, sitting on a park bench looking back on your life. What do you remember as the highlights?

Luciane engages in reflection informed by personal experiences and exposure to professional role models. She begins her day early, using quiet morning time to organize priorities and align daily activities with broader goals. She follows a structured weekly reflection cycle, setting goals at the beginning of the week and evaluating progress by the end to adjust strategies. When unexpected demands arise—such as grant‐driven priorities, service requests, leadership responsibilities, or complex decisions—she intentionally sets aside time, often during early weekend mornings, to realign priorities and respond proactively. This approach supports decision‐making in contexts where opportunities and career pathways may be constrained or unequally distributed. In such contexts, reflection becomes not only a planning tool, but a deliberate practice for navigating complexity and sustaining career progression.

Anton devotes two 30‐min sessions weekly to reflection. The first session is structured and practical: he reviews his calendar for the coming 2–3 weeks to adjust meetings; checks the status of ongoing projects; considers progress within his research team and schedules check‐ins as needed; and revisits monthly and annual goals, prioritizing those at risk of not meeting their timeline. The second session is more open: he reflects on problems or conflicts from the past week, considering how they might have been handled differently and what skills he might need to prevent their recurrence. If no urgent issues occurred, this time is used for broader reflection, guided by questions similar to those employed by Vin.

## 3 Clarify Your Vision, Missions for Each Life Role, and Actions to Achieve Them

The reflection process is an important initial step to allow you to create a vision and missions for each of your life roles (e.g., pharmacoepidemiologist, teacher, partner, parent, friend). You do not want to climb a mountain to perceived success only to determine later that you ascended the wrong mountain. To avoid that fate, you should use your reflection time to develop a vision for what the future you want to live in looks like for each role. You should consider creating for each of your roles a mission, which defines your purpose in that role and the current aims that you wish to achieve. A vision and missions will help you to focus on what is most important for success and satisfaction in your academic career and personal life [6].

Once you have drafted your vision and missions, you should next reflect on the goals needed to achieve your missions as well as the possible initiatives to take to meet those goals. It is often helpful to think backward from each mission to the actions needed instead of thinking forward. It may also be valuable to consider the actions that could lead to the opposite of what you seek and which you might want to avoid (a process called inversion [9]). For individuals navigating more dynamic or constrained environments, these actions may require more frequent reassessment and adaptation to respond to competing demands and evolving personal and professional circumstances. Examples of a vision, missions, and goals to advance the mission for selected life roles are included in Box 2.

BOX 2 **Example of a vision, mission, and goals to advance that mission for a pharmacoepidemiology researcher. Core values that undergird each mission are bolded.**

*Vision*: To generate research that is recognized as consequential, impactful, and significant, and helps to improve the health of patients and populations; and to provide teaching and mentorship that inspires and empowers my trainees to become the next generation of researchers.

*Mission*: To display **courage**, **curiosity**, **creativity**, and **caring** in leading multidisciplinary teams and educating/mentoring researchers at all stages to conduct rigorous, high‐quality pharmacoepidemiologic research using real‐world data to generate evidence to improve the health of patients and populations.

*Goals to Advance Mission*:

1. Schedule weekly mentor meetings in calendar with mentor.
2. Draft and send out purpose‐driven agendas for each research meeting at least 48 hours in advance of the meeting.
3. Identify items requiring action upon conclusion of each research meeting; distribute meeting minutes with action items (and individual assigned responsibility for completing the task) at least 48 hours after the meeting.
4. Prepare initial draft of abstract detailing the rationale, methods, key results, and conclusions of Aim 1 of project by December of current calendar year.
5. Prepare initial draft of the manuscript for Aim 1 of project by March of next calendar year.

### 3.1 Authors' Perspectives

Each of us has independently developed highly similar processes to create our vision, missions, and goals. Every December, we set aside time to audit the past year and update our values, vision, missions, goals, and initiatives—using the transition between years as a natural inflection point. This audit includes identifying activities to scale up or down, strengthening existing skills, addressing weaknesses, and setting metrics of success for the year ahead. In the final, quieter week of December, we review and finalize our plans, which then serve as a living document that we revisit during reflection periods throughout the year to stay aligned with our vision and actions.

## 4 Prioritizing Your Activities to Achieve Your Vision and Missions

You will be faced with many requests for your time throughout your academic career in pharmacoepidemiology. It is therefore crucial to prioritize the activities that will advance your professional and personal missions, consistent with Stephen R. Covey's third habit of highly effective people whereby one “puts first things first” and “keeps the main things the main things” [5]. Prioritizing your activities within the context of your goals and missions can be supported by considering the importance and urgency of tasks using the Eisenhower Matrix, a 2 × 2 grid with urgency on the y‐axis and importance on the *x*‐axis [10]. Visualizing where particular tasks lie on the Eisenhower Matrix can prompt which to focus on, which to delegate, and which to eliminate. Your mentors will often also be instrumental in reviewing your responsibilities (e.g., teaching, speaking, research collaborations, institutional service), deciding which to prioritize, and considering which, if any, could be phased out or eliminated. Mentors can also provide advice to help determine whether requests for your time can be declined.

### 4.1 Authors' Perspectives

At the start of each semester, Greta establishes clear goals and timelines for first and senior author research publications and conference submissions along with planning and preparations for upcoming grant applications. When considering new research projects or optional service requests, she evaluates where it fits within her larger mission and, importantly, where the time will come from, viewing work‐related time as a limited, fixed resource during her current phase of life. She considers the trade‐off of taking time and resources away from ongoing research or service activities for a new activity.

Luciane views prioritization as a dynamic process that requires a balance between strategic objectives and competing demands that are not always visible or formally acknowledged. She uses regular reflection to identify which activities should be maintained, adapted, or discarded, while remaining aligned with long‐term objectives. This approach emphasizes discipline, clarity of purpose, and the ability to make deliberate choices even in complex and constrained environments. This is an iterative process shaped by trial and error. In this context, the ability to prioritize is not just a productivity skill, but a critical component for sustaining a meaningful and equitable academic career.

Vin reviews his missions and goals regularly to remind him to avoid activities that will distract him from his vision. He has cultivated the habit of taking time on the weekend to identify and schedule his priorities for the upcoming week. This “weekly blueprint” ensures that the most important academic pursuits (e.g., continued education, grant writing, research meetings, manuscript preparation, data analysis, mentor meetings) and personal activities (e.g., reflection/journaling, connecting with family and friends, exercise) are prioritized and scheduled.

Anton has developed a full framework for prioritization based on eight specific tools (described in Box 3) to assess the value of a given task and guide which opportunities to accept and which to decline [11]. As examples, one tool scores the value of an opportunity across three domains (reward, collaborators, scientific value). A second tool prompts comparison of the new opportunity to already accepted and ongoing tasks. A third tool asks you to determine whether engagement in that opportunity will help you move closer to your vision. A recorded presentation of all eight tools can be found at: www.antonpottegaard.dk/JustSayNoVideo.

BOX 3 **Eight practical tools to help prioritize opportunities and determine which to accept and which to decline.**

**Table:** 

Tool to guide decision	Description
1. Mydtskov criteria	Named after a Danish jazz musician. To accept an opportunity, you should require that it meets *at least two* of the following criteria: (1) it will be fun, (2) it will offer great pay (e.g., funding, citations, etc.), and/or (3) it will allow you to work with great people. As an example, you could engage in a fun project with little pay if it allows you to work with great people, but you should avoid the project if it is boring with horrible pay, even if it allows you to work with wonderful people.
2. “72‐hour” rule	Before accepting a project, consider putting it on quarantine for 72 hours . If you remain enthusiastic about this opportunity 72 hours later, it might be worth pursuing. This tool is particularly important if you feel very excited about the opportunity (and thus might not be thinking clearly).
3. Tickbox optimization	Ask yourself how many items of importance (i.e., tickboxes) that you can check off if you pursue this opportunity. If the answer is only one or few, you might be better off pursuing an opportunity that addresses multiple items of importance.
4. JOMO!	“Joy (not fear) Of Missing Out.” Once an opportunity is turned down, set time aside to actively think about how happy you are now that you are not doing it. This activity can build confidence in future decisions.
5. Senior input	Discuss the opportunity with your peers or mentors. They can help determine what opportunities to accept and which to decline.
6. Vision	Ask yourself if this project moves you closer (or away from) your vision and/or mission. If you are about to invest your time in something that does not move you forward along the path you have chosen to prioritize, you should consider turning down the opportunity.
7. “To do” comparison	Before accepting a new project, review your current list of projects and your list of other new ideas. The new project offered to you might sound interesting, but you should ask yourself if it is more interesting or valuable than those that you are already pursuing. The new opportunity should be more interesting and valuable, since your other projects will inevitably suffer from the competition.
8. Constant reminder	We all need to become better at saving time for what's truly important to us, and many of us need to be reminded constantly. A picture on your desk or on your phone of the things that are most important to you, or other physical reminders or rituals, can serve as a constant reminder to follow your vision/missions.

## 5 Seek and Maintain Mentorship Throughout Your Career

Mentorship and feedback are essential throughout your career and should come from trusted individuals invested in your success [12]. Having multiple mentors is valuable since no single person can provide all needed expertise [13]. Mentors can help you clarify your vision and missions, prioritize actions, guide decisions about opportunities, and support work‐life balance. Potential mentors include research advisers, senior colleagues, peers, or others with relevant experience. While advisers and senior faculty often serve as mentors, peer mentorship can be particularly valuable for providing encouragement and emotional support, navigating programmatic expectations, and fostering professional identity. You may meet with mentors individually to discuss personal challenges, in pairs or small groups for diverse perspectives, or even convene your full group of mentors when collective input is helpful. During these meetings, you may wish to request sponsorship to further your development, such as presenting at conferences, receiving specialized training, or visiting external research teams. The format and frequency of meetings should be tailored to your needs and adjusted as your career and life circumstances evolve.

It is critically important to seek out and maintain mentorship throughout your career [14]. Although mentorship is often established during training, it frequently wanes after faculty appointments, even though guidance remains crucial for navigating decisions about opportunities, research focus, and promotion timing. As seniority and responsibilities grow, honest feedback becomes rarer and must be actively sought, making mentorship increasingly valuable over time.

Throughout each stage of your career, you should nurture the connections with each of your mentors and express gratitude for their contributions. Your mentors volunteer their time for you, and these relationships should not be simply transactional.

### 5.1 Authors' Perspectives

Each of us has received invaluable feedback from our mentors. At various stages of our academic career, we have maintained relationships with 3–5 mentors who have expertise in pharmacoepidemiology science, education/mentorship, team building, research dissemination and communication, leadership and management, and work‐life balance. Their input has enriched our day‐to‐day work experiences, improved our decision‐making and career choices, enhanced the rigor of our science, elevated the quality of our own mentorship, prompted consideration of leadership roles and the timing of these positions, and helped us navigate times with professional or personal challenges. Our mentors have also served as sponsors, recommending us as speakers at national and international meetings, as organizers of conferences, and for national and international awards.

## 6 Key Skills to Acquire and Benchmarks to Meet Across Your Academic Career

**FIGURE 1:**
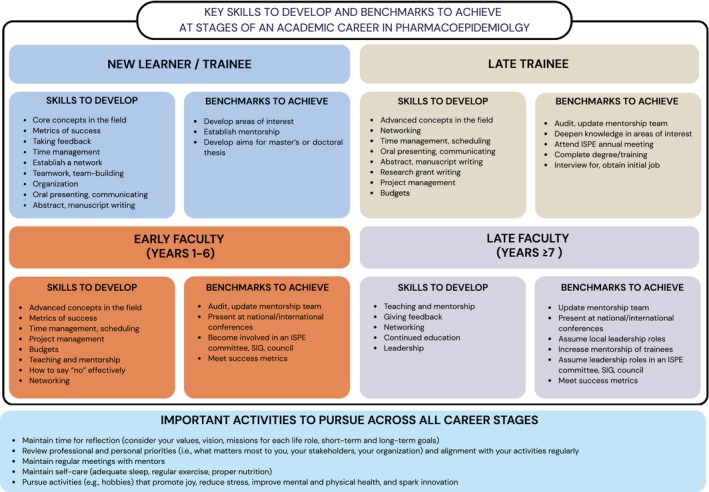
Key skills to develop and benchmarks to achieve at each specified stage of an academic career in pharmacoepidemiology. Abbreviations: ISPE = International Society for Pharmacoepidemiology; SIG = special interest group.

As academic pharmacoepidemiologists move through different stages of their career, they may need to develop key skills and achieve certain benchmarks to facilitate success (Figure 1). The skills we suggest developing during each career stage are recommendations and may occur earlier or later in a career depending on individual experiences. Many of these skills are not necessarily specific to our field, but proficiency in them could add substantial value to one's career and life. Importantly, while Figure 1 presents a structured progression of skills and benchmarks, career development is rarely linear. The acquisition of these competencies and achievement of milestones may be shaped by structural and contextual factors, including access to mentorship, resources, and opportunities. Individuals may develop skills at different times, revisit stages, or progress along non‐traditional paths depending on their circumstances.

### 6.1 New Learner/Trainee

New learners/trainees in a master's or doctoral program in pharmacoepidemiology will focus much of their time on learning core concepts in the field. Other important competencies for new learners/trainees to develop include time management and scheduling, organization, teamwork, and learning to respond constructively to feedback provided by mentors and instructors [15]. New learners/trainees should seek opportunities for oral presentation, abstract writing, poster presentation, and manuscript writing, which can provide valuable experience in research dissemination and communication. They should also consider establishing a support network of peers, which fosters a sense of community, bolsters learning of core competencies, and offers a safe space for emotional expression.

Benchmarks that should be met at this stage include developing core competencies in the field [16], establishing relationships with mentors [14], identifying areas of interest for study [17], and formulating aims for a master's or doctoral thesis [18, 19].

### 6.2 Late Trainee

Late trainees, who may be in the latter part of their doctoral program or engaged in a postdoctoral fellowship, should seek to develop approaches to cultivating new research ideas, learn advanced concepts used in their field of study [16], establish a network of colleagues who could potentially serve as mentors and collaborators, and potentially seek approaches to developing new research methods. They should continue to develop skills in time management and scheduling, oral presentation, abstract writing, poster presentation, and manuscript writing. As late trainees advance their research, they should seek training and experience in teaching, project management (particularly how to bring projects to completion effectively) [20], research grant writing [21], and developing budgets and justifications [22], which will be valuable for the transition to the early faculty stage. Late trainees should work closely with their mentors to set aside dedicated time for preparation and submission of research grants, which may be necessary for a faculty position at some institutions.

Benchmarks that late trainees should achieve include deepening knowledge in key areas of interest; auditing and updating their mentorship team; completing their degree or postdoctoral training; attending meetings of the International Society for Pharmacoepidemiology (ISPE) to observe rigorous pharmacoepidemiology methods, present research, and facilitate networking opportunities; and interviewing for and obtaining their initial job after training.

### 6.3 Early Faculty (Years 1–6)

With their transition to this position, early faculty members achieve independence and assume full responsibility for their time management and performance. Many are often unaware of the specific criteria on which they will be evaluated outside of the number or impact of original research articles, such as research funding, number of invited presentations, teaching requirements, and mentoring. Clarifying the metrics of evaluation at the outset, particularly by regular consultation with mentors, can help ensure that early faculty members meet their institution's priorities.

At this stage, it is important to continue to hone skills in team building and leadership, project management, and drafting budgets and justifications for projects. Early faculty should consider which tasks could be delegated to free up time to focus on higher‐value activities. If possible, they should try to minimize activities misaligned with their priorities and concentrate on those most critical to their vision and missions [23]. Early faculty should seek guidance from mentors to ensure sound decisions about their activities and requests for their time.

It is also critical to receive training and guidance in mentorship as well as teaching skills, including curriculum development, cultivating a positive classroom environment, listening, communication, and the ability to adapt to learners of different levels. Early faculty should try to develop a teaching portfolio specific to their areas of interest and seek feedback on their activities to facilitate improvement.

Benchmarks to achieve at this stage include reviewing and updating the mentorship team, identifying the metrics of success to meet on a semi‐annual and annual basis, increasing visibility and presentations at national and international conferences, and becoming involved in an ISPE committee, special interest group, or council.

### 6.4 Late Faculty (Years ≥ 7)

Late faculty members will increase their mentorship of trainees and early faculty [24, 25, 26]. To aid in this endeavor, late faculty should consider participating in workshops to grow their skills in guiding learners, delivering feedback effectively, and developing an authentic mentorship style.

At some point during this stage of their academic career, late faculty will likely be offered leadership or administrative roles, allowing them to have influence over the success of others. Late faculty should obtain honest feedback from mentors on the potential benefits and pitfalls of such positions, the appropriate timing to take on these roles, and important challenges and opportunities with the positions to facilitate optimal decision‐making. Late faculty should seek to enhance their leadership skills through the guidance of mentors and with workshops, self‐directed readings, and professional coaching. They should also conduct an audit of their activities to consider which should be reduced or phased out.

Benchmarks to meet at this stage include continuing to perform regular audits of the mentorship team and updating them with the necessary expertise, increasing mentorship of trainees and early faculty members, maintaining time for learning, and continuing to present at national and international conferences. Late faculty might also assume leadership or administrative roles within their organization and take on leadership roles in an ISPE committee, special interest group, or council.

### 6.5 Important Activities to Pursue Across All Career Stages

Regardless of career stage, it is crucial to maintain time for reflection, review personal and professional priorities, and assess the alignment of activities with priorities at least semi‐annually. Regular meetings with mentors should be scheduled. Adequate self‐care, such as sufficient sleep, regular exercise, and optimal nutrition, is also essential to help promote achievement of personal and professional priorities. Maintaining time to pursue hobbies can promote joy, reduce stress, improve mental and physical health, and spark innovation [27].

### 6.6 Navigating Transitions

While transitions are occurring across the career arc, moving to a different institution is common during an academic career. Transitions to different institutions may occur because of personal motivations (e.g., desire for closer proximity to family) or institutional reasons (e.g., lack of open position or leadership opportunity at the current institution). Transitions may also be sought to learn new skills, pursue new teaching or research opportunities, or seek a new leadership role. Mentors are instrumental in considering whether and how to transition to a different institution, and they can provide important feedback, since such actions can greatly affect academic career satisfaction and success.

## 7 Fostering Work‐Life Balance Throughout Your Career

Life events, such as pregnancy, becoming a parent, caregiving, loss of loved ones, or other personal responsibilities or challenges, can shift priorities and make it difficult to find time to reflect, clarify your priorities, learn new skills, and manage work responsibilities, impacting career trajectory [28]. To successfully navigate these events, it is critically important to: (1) build strong support networks, (2) leverage flexible work arrangements, and (3) set boundaries.

### 7.1 Building Support Networks

Connecting with others in academia can identify solutions that may alleviate tensions and help you adjust to changes that occur following major life events. It may be helpful to find mentors with similar lived experiences (e.g., marriage, parenting, care for older family members, divorce, death) to provide guidance on balancing work and personal responsibilities, along with advice on specific work‐related tasks.

### 7.2 Leveraging Flexible Work Arrangements

It may be helpful to seek out accommodations that allow flexible hours or remote work to accommodate life events such as childcare or illness. You should make yourself aware of institutional policies. Identifying and taking advantage of paid parental or medical leave, if available, can be valuable, if not essential.

### 7.3 Setting Boundaries

Setting boundaries on working hours, such as deciding how much time to dedicate to a specific task, establishing deadlines, and declining extra service tasks may help to promote balance that aligns with your current phase. Delegating tasks may be valuable to maintain productivity during demanding periods.

## 8 Summary

It is critically important to be proactive and intentional in designing your academic career in pharmacoepidemiology. Reflection is valuable to consider your values, vision, personal and professional priorities, missions for each role in your life, and short‐ and long‐term goals. Thoughtful prioritization of activities should be undertaken to maintain alignment with one's vision, missions, and goals. Mentors can provide useful advice and guidance to ensure that key skills are acquired and important benchmarks are met at each career stage. Such strategies can promote satisfaction and success in an academic career in pharmacoepidemiology.

We hope that our ideas will provide a framework that encourages reflection, goal setting, and intentional action that will help to overcome obstacles that may be encountered. By sharing our approaches and tools, we aim to stimulate conversations between trainees, faculty, mentors, and colleagues about how to design meaningful and sustainable careers. We believe that such discussions can ultimately strengthen the field and support the growth and satisfaction of its members.

## Funding

The authors have nothing to report.

## Conflicts of Interest

The authors declare no conflicts of interest.
